# Continental Nursing Institutions

**Published:** 1888-03-24

**Authors:** 


### CONTINENTAL NURSING INSTITUTIONS.

Mrs. Williams encloses the address of three continental nursing institutions for Nurse Johnson: Miss Pearson, Les Agaves, Cannes, France; Miss Woodcock, the Holland Institute of Nursing, Nice, France; The Home for Invalid Ladies, San Remo, Italy.

